# Clinical correlates of age at onset distribution in bipolar disorder: a comparison between diagnostic subgroups

**DOI:** 10.1186/s40345-017-0097-1

**Published:** 2017-08-21

**Authors:** Mirko Manchia, Giuseppe Maina, Bernardo Carpiniello, Federica Pinna, Luca Steardo, Virginia D’Ambrosio, Virginio Salvi, Martin Alda, Alfonso Tortorella, Umberto Albert

**Affiliations:** 1 Section of Psychiatry, Department of Medical Science and Public Health, University of Cagliari, Via Liguria, 13, 09127 Cagliari, Italy; 2 Department of Pharmacology, Dalhousie University, Halifax, NS Canada; 3 Department of Mental Health, “San Luigi-Gonzaga” Hospital, University of Turin, Orbassano, Italy; 4 Department of Psychiatry, University of Naples SUN, Naples, Italy; 5 Department of Psychiatry, Dalhousie University, Halifax, NS Canada; 6 Rita Levi Montalcini Department of Neuroscience, Anxiety and Mood Disorders Unit, University of Turin, Turin, Italy

**Keywords:** Mood disorders, Diagnostic subtypes, Early onset, Retrospective study, Admixture analysis

## Abstract

**Background:**

Admixture analysis of age at onset (AAO) has helped delineating the clinical profile of early onset (EO) bipolar disorder (BD). However, there is scarce evidence comparing the distributional properties of AAO as well as the clinical features of EO BD type 1 (BD1) with EO BD type 2 (BD2). To this end, we studied 515 BD patients (224 BD1, 279 BD2, and 12 BD not otherwise specified [NOS]) diagnosed according to DSM-IV-TR criteria.

**Methods:**

AAO was defined as the first reliably diagnosed hypo/manic or depressive episode according to diagnostic criteria. We used normal distribution mixture analysis to identify subgroups of patients according to AAO. Models were chosen according to the Schwarz’s Bayesian information criteria (BIC). Clinical correlates of EO were analysed using univariate tests and multivariate logistic regression models.

**Results:**

A two normal components model best fitted the observed distribution of AAO in BD1 (BIC = −1599.3), BD2 (BIC = −2158.4), and in the whole sample (BIC = −3854.9). A higher number of EO BD2 patients had a depression-(hypo)mania-free interval (DMI) course, while a higher rate of (hypo)mania-depression-free interval (MDI) course was found in EO BD1. EO BD2 had also a higher rate of comorbidity with alcohol dependence compared to EO BD1. The latter finding was confirmed by multivariate logistic regression analysis.

**Conclusions:**

In conclusion, both BD1 and BD2 had bimodal AAO distributions, but EO subgroups had a diagnostic-specific clinical delineation.

## Background

Bipolar disorder (BD) is a heritable psychiatric illness characterised by cyclic mood episodes of opposite polarity alternating with intervals of well-being (Goodwin and Jamison 2007). As in other psychiatric complex genetic diseases, the relatively high clinical heterogeneity of BD might have hindered the identification of molecular and clinical determinants of risk as well as of predictors of treatment outcome (Alda 2004). The magnitude of clinical heterogeneity might be reduced by studying subgroups of BD patients sharing specific clinical characteristics such as patterns of treatment response (Alda et al. 2005), mood incongruent psychosis (Goes et al. 2007), or early illness onset (Jamain et al. 2014). Indeed, the extensive analysis of age at onset (AAO) BD subgroups through admixture analysis has shown clinical (Bellivier et al. 2001; Lin et al. 2005; Manchia et al. 2008; Tozzi et al. 2011; Ortiz et al. 2011) and genetic (Etain et al. 2006; Severino et al. 2009; Etain et al. 2010; Belmonte et al. 2011) characteristics specific, particularly, to early onset (EO) BD.

As the vast majority of studies investigated BD type 1 (BD1) samples, it remains to be established, however, whether this clinical delineation of EO is present also in BD type 2 (BD2) patients. Furthermore, the distributional properties of AAO have never been investigated in samples exclusively composed of BD2 patients.

## Methods

### Aims

The primary aim of the present study was to test whether BD1 and BD2 differed in terms of AAO distributions. The secondary objective was to test whether EO had clinical characteristics specific for each diagnostic subgroup. To this end, we (i) studied the AAO distribution of each diagnostic subgroup with mixture modelling; (ii) compared the AAO distributions identified in BD1 and BD2; (iii) analyzed the pattern of associations of a set of demographic and clinical variables with EO in each diagnostic subgroup; and (iv) compared the clinical association patterns between EO BD1 and EO BD2.

### Patient population and assessment instruments

Our sample consisted of 515 unrelated patients with BD. Two hundred and twenty-four were diagnosed with BD1, while 279 had a diagnosis of BD2, and 12 had a diagnosis BD not otherwise specified (NOS). All subjects were of Italian ancestry. Patients were recruited at the Anxiety and Mood Disorders Unit, University of Turin, Italy and at the Department of Psychiatry, University of Naples SUN, Napoli, Italy. Certified psychiatrists with at least 4 years of postgraduate clinical experience performed the clinical assessment of patients. All potential interviewers met prior to study beginning and underwent a common extensive training prior to conducting the assessments. They were trained in the use of a common semi-structured interview that was used to collect (a) socio-demographic data (age, gender, marital status, years of education, and occupational status); (b) diagnosis (current and lifetime), which were performed according to the Diagnostic and Statistical Manual of Mental Disorders (DSM)-IV-Text Revision (TR) criteria (American Psychiatric Association 2000) using the structured clinical interview for DSM-IV-TR Axis I disorders (SCID-I/P) (First et al. 2002); (c) clinical data including AAO. In addition, a systematic review of patients’ medical records helped clinicians to establish AAO and corroborate data concerning clinical characteristics of the disorder emerging from direct interview. Age at onset was defined as the first reliably diagnosed hypo/manic or depressive episode meeting the diagnostic criteria. External corroboration for AAO was obtained, whenever possible, by directly interviewing, with patient’s consent, a first-degree family member or other significant individuals. For the purposes of the present study, we included only subjects for whom it was possible to establish AAO with complete agreement between the information provided by patients and their relatives. Age at interview was defined as the age at which subjects were first assessed by a clinician at each research centre.

In the early phase of the study, inter-rater reliability of the diagnosis of Axis I disorders with the SCID-I was ascertained. The inter-rater reliability was found to be good: Cohen kappa coefficient was 0.89 for the presence of any current or lifetime Axis I disorder.

### Data analysis

We used Gaussian distribution mixture analysis to test whether we could identify subgroups of patients according to the AAO. We investigated a range of number of AAO groups (1–9). The choice of the mixture model that best fit the distribution of AAO was made according to the Schwarz’s Bayesian information criteria (BIC). Specifically, the analysis performed with the “Mclust” (Fraley and Raftery 1999; Fraley et al. 2014) package implemented in R (R Development Core Team 2008) indicates the best model as the one with the highest BIC among the fitted models (Fraley and Raftery 2007). This package estimates parameters of the model using an expectation–maximization (EM) algorithm. Cut-off points were derived using the Gaussian cumulative distribution function of estimated AAO mixture function and calculating each data point’s probability of belonging to each class. Specifically, once the mixture model parameters were estimated, we calculated the posterior probability of any data point. The resulting probabilities were then compared in order to establish which class the data point belonged to. Gaussian mixture analysis (both number of components and parameters estimates) was also replicated and confirmed with the “Mixtools” R package (Benaglia et al. 2009). We used Kolmogorov–Smirnov (K–S) test to determine whether the Gaussian cumulative distribution function of estimated AAO mixture identified in BD1 patients was significantly different from the one identified in BD2 patients. Further, we used K–S to test for differences in the AAO Gaussian cumulative distribution function of estimated AAO mixture between participating research centres. We tested the association of continuous and categorical clinical variables with AAO subgroups using univariate analysis (*t* test or *χ* ^*2*^ test as appropriate). The independent variables tested included sex, age at interview, diagnosis, illness duration, presence of family history of any DSM-IV-TR psychiatric disorder, presence of family history of BD and any DSM-IV-TR mood disorders, number of manic/hypomanic, depressive and mixed episodes, type of clinical course cycle [i.e. (hypo)mania-depression-free interval (MDI), depression-(hypo)mania-free interval (DMI), irregular cycling, continuous cycling, rapid cycling], presence of lifetime suicidal behaviour, lifetime comorbidity with substance (other than alcohol) dependence, and lifetime comorbidity with drug and/or alcohol dependence. Statistical significance was set at *α* = 0.05. Only clinical variables presenting a statistical significant association with an AAO subgroup (*p* < 0.05) of each diagnostic sample (BD1 and BD2) were entered into a backward stepwise multivariate binary logistic model to account for possible intercorrelations. All statistical analyses, except for mixture modelling, were performed with STATA/SE 12.0.

## Results

### Age at onset distribution: Gaussian mixture analysis

**Fig. 1:**
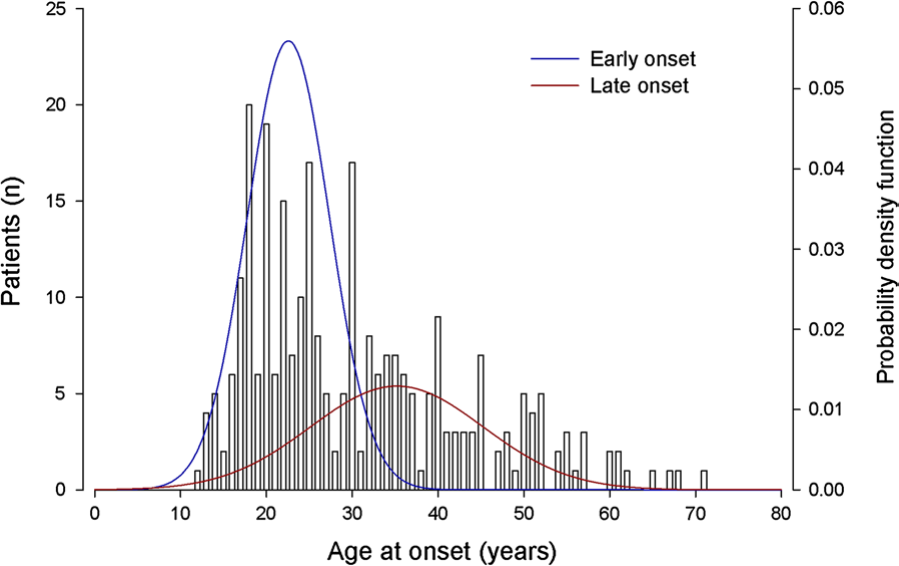
Age at onset distribution in bipolar disorder type 1 patients (*N* = 224). Gaussian probability density function was derived by the estimated age at onset mixture function in bipolar disorder type 1 patients

The BD1 sample (99 males and 125 females) had a mean age at interview (±SD) of 47.2 years (±13.0) and a mean AAO of 26.7 years (±9.2). A two normal components model best fitted the observed distribution of AAO (BIC = −1599.3) (Fig. 1). Models with three and four components did not improve the fit (Table 1).

**Table 1:** Age at onset distributions identified by Gaussian mixture analysis in bipolar disorder type 1 and type 2 and in the whole sample

Mixture model	BIC	Model components	Mean	SD	Proportion (%)
Bipolar disorder type 1 sample (*n* = 224)					
M1	−1637.7	1st component	26.7	9.1	100.0
*M2*	*−1599.4*	*1st component*	*22.6*	*4.8*	*66.9*
		*2nd component*	*35.1*	*10.1*	*33.1*
M3	−1611.0	1st component	18.7	3.0	27.1
		2nd component	25.6	4.4	45.7
		3rd component	36.6	10.1	27.2
M4	−1624.0	1st component	17.9	2.6	23.1
		2nd component	23.8	3.1	28.2
		3rd component	28.8	5.9	33.4
		4th component	40.9	9.9	15.3
Bipolar disorder type 2 sample (*n* = 279)					
M1	−2218.4	1st component	30.6	12.6	100.0
*M2*	*−2158.4*	*1st component*	*20.9*	*4.1*	*44.0*
		*2nd component*	*38.2*	*11.8*	*56.0*
M3	−2163.2	1st component	19.3	3.3	33.1
		2nd component	28.4	6.1	33.7
		3rd component	44.0	11.0	33.2
M4	−2177.7	1st component	17.6	2.5	22.8
		2nd component	23.0	2.8	24.7
		3rd component	32.2	4.4	22.7
		4th component	45.6	10.3	29.8
Total bipolar disorder sample (*n* = 515)					
M1	−3986.7	1st component	29.0	11.5	100.0
*M2*	*−3854.9*	*1st component*	*21.9*	*4.6*	*55.0*
		*2nd component*	*37.6*	*11.5*	*45.0*
M3	−3856.0	1st component	19.2	3.2	28.9
		2nd component	26.5	5.6	40.6
		3rd component	41.5	11.2	30.6
M4	−3868.3	1st component	17.8	2.6	22.8
		2nd component	23.3	3.1	28.0
		3rd component	31.1	5.1	24.0
		4th component	43.4	10.9	25.2

Best fitting mixture models are typed in italics

*M* model, *BIC* Bayesian information criteria, *SD* standard deviation

The EO component had a mean AAO of 22.6 years (±4.8), while the late onset (LO) component had a mean AAO of 35.1 years (±10.1) comprising 67% and 33% of the population proportion, respectively. The cut-off point, derived by the Gaussian cumulative distribution function of the latter estimated AAO function, was at 32 years for BD1 (EO group <32 years; LO group ≥32 years) with 169 patients in the EO group and 55 in the LO group.

**Fig. 2:**
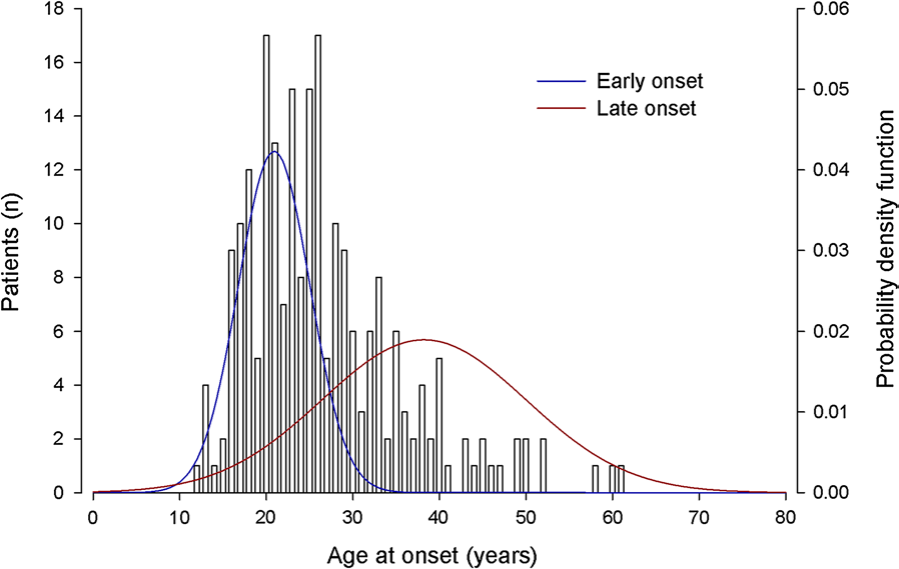
Age at onset distribution in bipolar disorder type 2 patients (*N* = 279). Gaussian probability density function was derived by the estimated age at onset mixture function in bipolar disorder type 2 patients

The BD2 sample (114 males and 165 females) had a mean age at interview of 50.6 years (±14.8) and a mean AAO of 30.6 years (±12.7). The observed distribution of AAO was also best fitted by a two normal components model (BIC = −2158.4) (Fig. 2).

No improvement of the fit was observed with three and four components models (Table 1). The BD2 sample had an EO component with mean AAO of 20.9 years (±4.1) and a LO component with mean AAO of 38.2 years (±11.8) with population proportions of 44% and 56%, respectively. The cut-off point, derived by the Gaussian cumulative distribution function of this estimated AAO function, was at 28 years (EO group <28 years; LO group ≥28 years). The EO group comprised 142 patients, while the LO group included 137 patients.

**Fig. 3:**
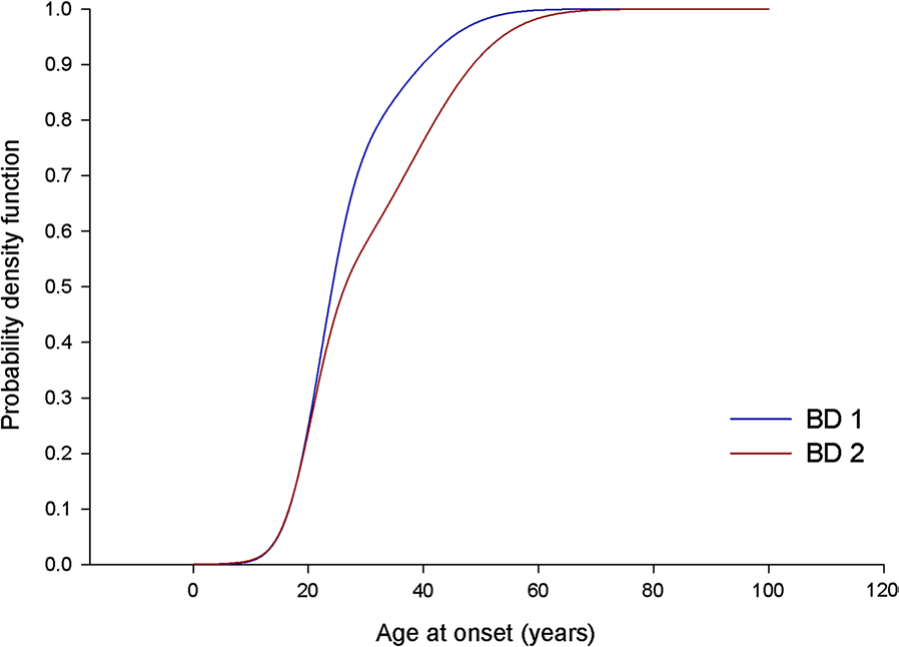
Kolmogorov–Smirnov test between theoretical age at onset in bipolar disorder type 1 and type 2 patients. Probability density functions of the two estimated age at onset mixture function. *BD1* bipolar disorder type 1, *BD2* bipolar disorder type 2

**Fig. 4:**
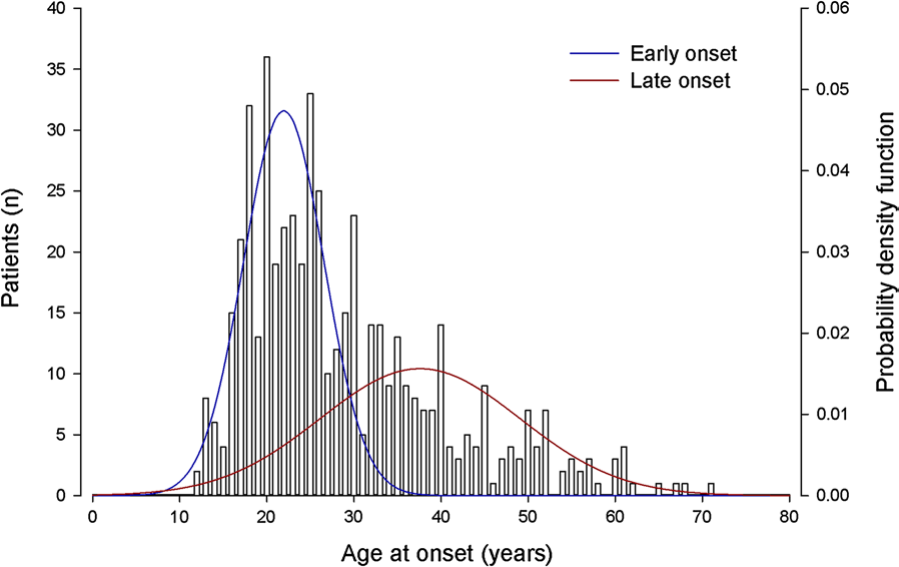
Age at onset distribution in the whole sample of bipolar disorder patients (*N* = 515). Gaussian probability density function was derived by the estimated age at onset mixture function in the whole sample of bipolar disorder patients

Kolmogorov–Smirnov test showed that the Gaussian cumulative distribution functions of estimated AAO mixture of BD1 and BD2 differed significantly (*D* = 0.18, *p* < 0.00001) (Fig. 3). Conversely, there was no significant difference between the Gaussian cumulative distributions of estimated AAO mixture of the two participating research centres (*D* = 0.05, *p* = 0.57). Finally, Gaussian mixture analysis confirmed a best fitting model of two normal components (detailed in Table 1) in the whole sample of 515 BD patients (216 males and 299 females), which included 12 subjects with BD NOS (Fig. 4). The cut-off point, derived by the Gaussian cumulative distribution function of this estimated AAO function, was at 30 years.

### Clinical correlates of early onset: patterns of association in bipolar disorder type 1 and type 2 diagnostic subgroups

As shown in Table 2, a trend association (*p* = 0.05) was identified for the presence of family history of any DSM-IV-TR mood disorder, with LO BD1 having a higher rate than EO BD1. Early onset BD1 had a lower age at interview and a longer duration of illness than LO BD1.

**Table 2:** Comparison of clinical correlates between early and late onset bipolar disorder type 1 and type 2 patients

Clinical variable	Bipolar disorder type 1 (*N* = 224)		*χ* ^*2*^ or *t*	*P*
	Early onset (*N* = 169)	Late onset (*N* = 55)		
Female (%)	73 (43.2)	26 (47.3)	0.3	0.6
Age at interview, mean (SD)	45.3 (13.5)	53.1 (8.8)	4.0	*<0.0001*
Presence of family history of any psychiatric disorder (%)^a^	99 (58.9)	37 (67.3)	1.2	0.3
Presence of family history of mood disorder (%)^b^	85 (52.5)	34 (68.0)	3.7	0.05
Presence of family history of bipolar disorder (%)^c^	31 (18.5)	13 (23.6)	0.7	0.4
Number of manic/hypomanic episodes, mean (SD)^d^	4.4 (5.1)	3.6 (3.2)	−1.1	0.3
Number of depressive episodes, mean (SD)^e^	5.5 (4.7)	4.8 (4.4)	−0.9	0.3
Number of mixed episodes, mean (SD)^f^	0.6 (1.6)	0.8 (1.3)	0.8	0.4
Illness duration, mean (SD)	22.5 (12.8)	13.5 (8.6)	−4.9	*<0.0001*
Lifetime comorbidity with substance dependence (%)	15 (8.9)	4 (7.3)	0.1	0.7
Lifetime comorbidity with alcohol dependence (%)	10 (5.9)	1 (1.8)	1.5	0.2
Presence of suicidal behaviour (%)	47 (28)	11 (20)	1.4	0.2
Type of clinical course cycle				
MDI (%)	75 (44.4)	22 (40.0)	3.7	0.45
DMI (%)	20 (11.8)	11 (20.0)		
Irregular cycling (%)	65 (38.5)	21 (38.2)		
Continuous cycling (%)	5 (3.0)	1 (1.8)		
Rapid cycling (%)	4 (2.4)	0 (0.0)		

Clinical variable	Bipolar disorder type 2 (*N* = 279)		*χ* ^*2*^ or *t*	*p*
	Early onset (*N* = 142)	Late onset (*N* = 137)		
Female (%)	85 (59.9)	80 (58.4)	0.06	0.8
Age at interview, mean (SD)	42.8 (14.4)	58.7 (10.1)	10.6	*<0.0001*
Presence of family history of any psychiatric disorder (%)	93 (65.5)	87 (63.5)	0.1	0.7
Presence of family history of mood disorder (%)^b^	84 (60.0)	79 (58.5)	0.06	0.8
Presence of family history of bipolar disorder (%)	36 (25.4)	19 (13.9)	5.8	*0.02*
Number of manic/hypomanic episodes, mean (SD)^d^	4.3 (4.2)	3.7 (4.3)	−1.1	0.3
Number of depressive episodes, mean (SD)^e^	6.0 (5.6)	5.4 (5.3)	−1.0	0.3
Illness duration, mean (SD)^g^	22.0 (13.7)	17.8 (11.2)	−2.8	*0.005*
Lifetime comorbidity with substance dependence (%)	8 (5.6)	6 (4.4)	0.2	0.6
Lifetime comorbidity with alcohol dependence (%)	19 (13.4)	6 (4.4)	6.9	*0.01*
Presence of suicidal behaviour (%)	45 (31.7)	29 (21.2)	3.9	0.06
Type of clinical course cycle				
MDI (%)	51 (35.9)	45 (32.8)	3.9	0.4
DMI (%)	31 (21.8)	35 (25.5)		
Irregular cycling (%)	58 (40.8)	52 (38.0)		
Continuous cycling (%)	2 (1.4)	2 (1.5)		
Rapid cycling (%)	0 (0.0)	3 (2.2)		

Significant values are typed in italics

*MDI* (hypo)mania-depression-free interval, *DMI* depression-(hypo)mania-free interval, *SD* Standard Deviation, *p* p value

^a^ BD1: missing data for 1 patient

^b^ BD1: missing data for 12 patients, BD2: missing data for 4 patients

^c^ BD1: missing data for 1 patient

^d^ BD1: missing data for 1 patient, BD2: missing data for 4 patients

^e^ BD1: missing data for 1 patient, BD2: missing data for 4 patients

^f^ BD1: missing data for 1 patient, BD2: missing data for 3 patients

^g^ BD2: missing data for 2 patients

Early onset BD2 had a higher rate of comorbidity with alcohol dependence, as well as a higher rate of family history of BD in EO BD2 compared to LO BD2 (Table 2). Further, they also had a lower age at interview and longer illness duration than LO BD2.

We then performed a multivariate logistic regression in BD2 confirming that family history of BD [odds ratio (OR) = 1.97, 95% confidence interval (CI) 1.04–3.73, *p* = 0.04], comorbidity with alcohol dependence (OR = 3.2, 95% CI 1.21–8.54, *p* = 0.02), and illness duration (OR = 1.03, 95% CI 1.008–1.048, *p* = 0.006) were associated with EO. As age at interview was used to calculate illness duration, and consequently significantly correlated with it (*r* = −0.97), it was not included in the logistic regression model. Multivariate analysis was not performed in BD1 since no clinical variable was significantly associated with AAO subgroup in univariate test.

### Clinical correlates of early onset: comparison between bipolar disorder type 1 and type 2 diagnostic subgroups

The mean AAO was significantly lower in BD1 compared to BD2 (26.7 ± 9.2 vs. 30.6 ± 12.7; *t* = −3.8; *p* < 0.0001). The results of the univariate analysis are shown in Table 3. Early onset BD1 patients were older than EO BD2. Conversely, EO BD2 had a higher rate of comorbidity with alcohol dependence. In addition, a higher number of EO BD2 presented with a DMI course, while a higher rate of MDI course was found in EO BD1. The multivariate binary logistic regression confirmed the association of comorbidity with alcohol dependence with EO BD2 (OR = 0.4, 95% CI 0.18–0.90, *p* = 0.02).

**Table 3:** Comparison of clinical correlates between early onset bipolar disorder type 1 and type 2 patients

Clinical variable	Early onset diagnostic subgroup		*χ* ^*2*^ or *t*	*P*
	Bipolar disorder type 1 (*N* = 169)	Bipolar disorder type 2 (*N* = 142)		
Female (%)	96 (56.8)	85 (59.9)	0.3	0.6
Age at interview, mean (SD)	45.3 (13.5)	42.8 (14.4)	1.5	*0.01*
Presence of family history of any psychiatric disorder (%)	99 (58.9)	93 (65.5)	1.4	0.2
Presence of family history of mood disorder (%)	85 (52.5)	84 (60.0)	1.7	0.2
Presence of family history of bipolar disorder (%)	31 (18.5)	36 (25.4)	2.2	0.1
Number of manic/hypomanic episodes, mean (SD)	4.4 (5.1)	4.3 (4.2)	0.2	0.8
Number of depressive episodes, mean (SD)	5.5 (4.7)	6.0 (5.6)	−0.8	0.4
Illness duration, mean (SD)	22.5 (12.8)	21.9 (13.7)	0.4	0.7
Lifetime comorbidity with substance dependence (%)	15 (8.9)	8 (5.6)	1.2	0.3
Lifetime comorbidity with alcohol dependence (%)	10 (5.9)	19 (13.4)	5.1	*0.02*
Presence of suicidal behaviour (%)	47 (28.0)	45 (31.7)	0.5	0.5
Type of clinical course cycle				
MDI (%)	75 (44.4)	51 (35.9)	10.4	*0.035*
DMI (%)	20 (11.8)	31 (21.8)		
Irregular cycling (%)	65 (38.5)	58 (40.8)		
Continuous cycling (%)	5 (3.0)	2 (1.4)		
Rapid cycling (%)	4 (2.4)	0 (0.0)		

Significant values are typed in italics

*MDI* (hypo)mania-depression-free interval, *DMI* depression-(hypo)mania-free interval, *SD* Standard Deviation, *p* p value

## Discussion

The present study highlighted that a two normal component model in BD1 as well as in BD2 diagnostic subgroup best described the distribution of AAO. This finding was not reflected, however, in similar distributional properties of AAO, as well as in comparable pattern of association with clinical variables between the two diagnostic subgroups. In fact, our study found that EO BD2 patients had a higher rate of alcohol dependence compared to both LO BD2 and EO BD1. Further, EO BD2 patients had more frequently a DMI type of clinical course, while the MDI type was more frequently associated with EO BD1. Finally, EO BD2 showed a higher familial load for BD compared to LO BD.

The bimodal AAO distribution found in both BD diagnostic subgroups and in the whole sample of 515 patients is consistent with some studies (Ortiz et al. 2011; Kennedy et al. 2005; Javaid et al. 2011). Conversely, the majority of studies on mixture analysis of AAO showed a trimodal distribution in samples comprising mainly BD1 patients (Bellivier et al. 2001, 2003; Lin et al. 2006; Severino et al. 2009; Hamshere et al. 2009; Tozzi et al. 2011; Bellivier et al. 2014; Golmard et al. 2015). Of note, a recent study showed that bimodal and trimodal distribution fit equally well the AAO of BD (Grigoroiu-Serbanescu et al. 2014). Further research is needed to determine which distribution (bi- or tri-modal) better describes AAO in BD and which is (or which are) the best cut-off(s) before investigating clinical correlates and genetic differences between subgroups based on AAO. In fact, thresholds between subgroups found in different studies differed [e.g. thresholds between the intermediate and late AAO subgroups differed from 25 in one study (Tozzi et al. 2011) to 40 years in another (Hamshere et al. 2009)] as well as percentages of patients in each AAO subgroups [e.g. percentages of patients attributed to the early onset subgroup varied between 21.4% (Bellivier et al. 2001) and 79.7% (Lin et al. 2006)]. Discrepancies in the identified AAO distributions, cut-off points, and proportions of patients in each AAO subgroups may depend on diverse assessment methods, recall bias, study design (Montlahuc et al. 2016), and differences in characteristics of samples studied, including geographic location (Post et al. 2008; Bellivier et al. 2014) and birth cohort (Bauer et al. 2015; Golmard et al. 2015). Concerning study design, Montlahuc et al. (2016) tested whether cross‐sectional designs (which cause right truncation), unreliable diagnosis for individuals younger than 10 years old (which causes left truncation), and the selection criterion used for admixture analysis impacted the number of identified AAO subgroups. Importantly, a combination of left and right truncation, which is common in previously published studies of AAO admixture analysis, appeared to significantly influence the number of AAO subgroups detected (Montlahuc et al. 2016). Geographical location appears also to impact on AAO admixture analysis findings. Bellivier et al. (2014) found significant differences in the theoretical AAO functions between USA and European BD samples, mainly led by the higher proportion of patients in the EO subgroup and the lower mean AAO in the USA sample. Finally, birth cohort effect might also influence the estimation of AAO subgroups parameters. In this regard, Golmard et al. (2015) found that the proportion of EO cases increased substantially among BD cases born after 1960 compared to those born before the same year.

Several other findings deserve a comment. In our sample, BD type 1 patients had an earlier mean AAO (26.7 years) than BD2 patients (30.6 years), in agreement with existing data showing that BD1 first manifest their symptoms at an earlier age (Merikangas et al. 2011). In keeping, admixture analyses indicated a larger proportion of EO cases among BD1 patients (67%) compared to BD2 patients (44%). As a consequence, the EO BD1 group had a later mean AAO (22.6 years), compared to EO BD2 patients (20.9 years), reflecting in a higher AAO cut-off point (32 years for BD1 and 28 years for BD2).

These distributional properties of AAO distinguishing EO BD1 from EO BD2 resulted also in diverse patterns of clinical correlates of EO. Indeed, EO BD2 patients had a higher rate of alcohol dependence compared to both LO BD2 and EO BD1. Similarly, previous studies investigating clinical correlates of AAO subgroups found that EO BD patients have higher rates of alcohol abuse (Javaid et al. 2011; Lin et al. 2006). However, these studies either analysed only BD1 individuals (Lin et al. 2006) or did not specify the diagnostic stratification (Javaid et al. 2011). Interestingly, a recent study from Propper et al. (2015) did not find differences in rates of alcohol abuse among AAO subgroups in a sample with a BD1:BD2 ratio of 2:1.

Early onset BD2 patients had more frequently a DMI type of clinical course, while the MDI type was more frequently associated with EO BD1. Although not directly comparable with our study, the findings reported by Perlis et al. (2004) and Propper et al. (2015) in their samples relatively balanced in terms of BD1:BD2 ratio, indicated that very EO and EO BD patients have more frequently onset episodes of depressive polarity compared to later onset subgroups. In addition, our findings were consistent with the work of Koukopoulos et al. (2013), which found that patients with a DMI illness course are more likely to be BD2, while MDI illness course is overrepresented in BD1 patients.

Differently from what reported in the literature, our study failed to confirm in the BD1 subgroup the well-established association of EO BD with a higher familial load for BD and for mood disorders in general, as well as with higher rates of suicidal behaviour (Geoffroy et al. 2013). On the contrary, EO BD2 showed a higher familial load for BD (*p* = 0.02) compared to LO BD. Finally, although not statistically significant, EO BD2 showed higher rates of family history for BD as well as for mood disorders, compared to EO BD1. Similarly, Baek et al. (2011) found higher rates of major depression, but not of BD, in BD2 patients compared to BD1, although their sample was not stratified according to AAO. Further, another recent study showed that both BD1 and BD2 presented a similar familial load for mood disorders (Dell’Osso et al. 2016)

There is compelling evidence that EO BD patients appear also to be more frequently associated with rapid cycling, drug abuse, higher rates of obsessive–compulsive disorder, and possibly for psychotic features, and panic disorder (Geoffroy et al. 2013). Although our study did not test for association most of these clinical correlates, there were no statistically significant associations with EO for drug dependence. Of note, most of this evidence is derived from BD1 samples as there is a lack of data on the analysis of AAO in BD2, while only a few studies investigated mixed samples with both BD1 and BD2 patients (Perlis et al. 2004; Severino et al. 2009; Tozzi et al. 2011; Ortiz et al. 2011; Propper et al. 2015).

Our results should be interpreted in the context of some limitations. The retrospective assessment of AAO might have been subject to recall bias. However, data were gathered through direct interview of the patients as well as with systematic review of medical charts decreasing the probability of a systematic bias in the assessment of AAO. An additional limitation is the lack of a systematic approach in collecting family history data, which might have influenced the assessment of familial load in our sample. Moreover, external corroboration for AAO was obtained, whenever possible, by directly interviewing a first-degree family member or other significant individuals. Further, our samples of BD1 and BD2 patients might not have had an adequate statistical power to detect association signals of small to moderate magnitude. Finally, our study did not consider birth cohort effect in our analysis.

## Conclusions

To our knowledge, this is the first study specifically aimed at comparing clinical correlates of EO between BD1 and BD2 patients’ populations using admixture analysis. Our work found that, beside diverse distributional properties of AAO, BD1 and BD2 EO subgroups differed also in their clinical characteristics. Of note, our study identified a subgroup of EO BD2 with an AAO even earlier than in the EO BD1 subgroup, characterised by a higher genetic load (i.e. higher familial load for BD) and at greater risk of developing alcohol dependence. Should our findings be replicated in other studies directly comparing AAO BD1 subgroups with AAO BD2 subgroups, future work will be able to focus also on the differences in the genetic and biological makeup, possibly facilitating the search for reliable disease biomarkers.

## Abbreviations

BD: bipolar disorder
BD1: BD type 1
BD2: BD type 2
AAO: age at onset
EO: early onset
LO: late onset
NOS: not otherwise specified
BIC: Schwarz’s Bayesian information criteria
DMI: depression-(hypo)mania-free interval
MDI: (hypo)mania-depression-free interval
SCID-I/P: structured clinical interview for DSM-IV-TR Axis I disorders
KS: Kolmogorov–Smirnov
